# Antioxidant and Anti-Urolithiatic Activity of Aqueous and Ethanolic Extracts from *Saussurea costus* (Falc) Lispich Using Scanning Electron Microscopy

**DOI:** 10.3390/life12071026

**Published:** 2022-07-11

**Authors:** Naima Mammate, Fatima Ezzahra El oumari, Hamada Imtara, Salim Belchkar, Anissa Lahrichi, Ali S. Alqahtani, Omar M. Noman, Mahmoud Tarayrah, Tarik Sqalli Houssaini

**Affiliations:** 1 Laboratory of Epidemiology and Research in Health Sciences, Faculty of Medicine and Pharmacy, Dental Medicine, University Sidi Mohammed Ben Abdellah, BP 1893, Km 22, Road of Sidi Harazem, Fez 30070, Morocco; naima.mammate@usmba.ac.ma (N.M.); fatimezzahraeloumari@gmail.com (F.E.E.o.); salim.belchkar@usmba.ac.ma (S.B.); tarik.sqalli@usmba.ac.ma (T.S.H.); 2 Faculty of Arts and Sciences, Arab American University Palestine, P.O. Box 240, Jenin 44862, Palestine; 3 Laboratory of Biochemistry Faculty of Medicine and Pharmacy, Dental Medicine, University Sidi Mohammed Ben Abdellah, BP 1893, Km 22, Road of Sidi Harazem, Fez 30070, Morocco; anissa.lahrichi@usmba.ac.ma; 4 Department of Pharmacognosy, College of Pharmacy, King Saud University, Riyadh 11451, Saudi Arabia; alalqahtani@ksu.edu.sa (A.S.A.); onoman@ksu.edu.sa (O.M.N.); 5 Groupe Hospitalier Cochin-Port Royal, Faculty of Medicine, Institute Cochin, CNRS, IN-SERM, Paris University, 75000 Paris, France; mahmoud.tarayrah@hotmail.com

**Keywords:** calcium oxalate, cystine, FT-IR, *Saussurea costus* (Falc) Lipsch and SEM-EDX

## Abstract

The plant *Saussurea costus* (Falc) Lipsch has many biological activities and a strong curative and preventive power against a variety of diseases including cancer, diabetes, and hemorrhoids. In the current study, phytochemical screening was carried out as well as an investigation of the antilithiatic and antioxidant activities of aqueous and ethanolic extracts of this plant. The results showed that aqueous and ethanolic extracts were effective in reducing cystine stone mass and that the aqueous extract of *Saussurea costus* (Falc) Lipsch had the highest percentage of dissolution (6.756 ± 1.024) (*p* < 0.05). A turbidimetric method and a crystallization test were used to evaluate the antilithiatic activity of an aqueous and ethanolic extract of this plant on calcium oxalate crystallization. The results of these methods revealed that the ethanolic extract of this plant has a significant inhibitory effect on calcium oxalate crystallization, with a percentage inhibition of (91.017 ± 0.299) (*p* < 0.05) for a concentration of 2 mg mL^−1^. The DPPH method revealed that the ethanolic extract of *Saussurea costus* (Falc) Lipsch with a concentration of (IC50 = 0.12325 mg mL^−1^) had the highest IC50, whereas the FRAP method revealed that the aqueous extract of *Saussurea costus* (Falc) Lipsch with a concentration of 300 µg mL^−1^ has the most significant reducing power with (OD = 0.56 ± 0.05). These findings indicate that aqueous and ethanolic extracts of *Saussurea costus* (Falc) Lipsch had a significant effect on whewellite and weddellite and a greater free radical scavenging effect but had no effect on cystine dissolution.

## 1. Introduction

Urolithiatic disease is a worldwide public health problem that affects about 7% of women and 13% of men [1]. Kidney stones are mineral deposits that form inside the kidneys and are most commonly formed when urine becomes concentrated [2], allowing the minerals to crystallize and stick together. The etiopathogenesis of this disease is multifactorial, involving anatomical, environmental, genetic, and nutritional factors [3]. Furthermore, urolithiatic disease can be treated with techniques such as extracorporeal shock wave lithotripsy, percutaneous nephrolithotomy, and ureteroscopy [4,5,6]. Medication can also be used to treat it, but this is a time-consuming procedure with side effects, and the high cost of conventional therapies makes herbal therapy a promising alternative. More than 80% of kidney stones are composed of calcium oxalate [7,8], while 1% are composed of cystine, which is found in a small percentage of rarer kidney stones [9,10]. Due to the high cost and side effects of conventional treatment [11], the global public is becoming more interested in the use of herbal remedies in the treatment of urolithiatic disease [12].

The mechanisms underlying urinary stone formation remain unknown [13,14]. Crystal nucleation, aggregation, and growth of insoluble particles are thought to cause urinary lithiasis [2,15]. The damage that is caused by stone formation to the renal membrane is mediated by free radicals which facilitate crystalline retention on the papillary surface. Antioxidants, on the other hand, play a critical role in preventing kidney stone formation by maintaining a normal physiological concentration of free radicals [16,17].

The medicinal plant *Saussurea costus* (Falc) Lipsch was chosen to treat lithogenesis [18] as it contains chemical compounds that are known to be effective against urinary stones such as saponins [19] and flavanols. The latter is a subclass that contains many phytochemicals such as quercetin [20] and its derivatives (quercetin glycuronide, quercetin pentocide, and quercetin hexoside) [21]. This plant has been used in traditional medicine to treat kidney stones [18,22]. Furthermore, *Saussurea costus* (Falc) Lipsch is widely used in traditional medicine to treat a wide range of ailments. Various pharmacological experiments have conclusively demonstrated *Saussurea costus* (Falc) Lipsch’s ability to exhibit anti-inflammatory, anti-ulcer, anti-cancer, and hepatoprotective activities [21], which justifies its traditional uses [20,21,22]. The purpose of this study is to evaluate the antilithiatic activity of aqueous and ethanolic extracts of this plant. As a result, we used a scanning electron microscope (SEM) in conjunction with EDX to track the variation of cystine stone mass and structural changes before and after treatment [8,23]. This research was conducted to provide information of phytochemical screening and the antilithiatic activity of aqueous and ethanolic extracts of *Saussurea costus* (Falc) Lipsch against calcium oxalate crystallization using turbidity and crystallization tests. In addition, the antioxidant activity of the various extracts that were studied was determined using DPPH and FRAP to determine their antiradical and reducing power.

## 2. Materials and Methods

### 2.1. Sample Preparation and Extraction

The roots of *Saussurea costus* (Falc) Lipsch were purchased from the local market in Fez, Morocco, and it was exported from India on 15 March 2021. The specimens were kept at the Laboratory of Epidemiology and Research in Health Sciences, Faculty of Medicine, Pharmacy and Dental Medicine, University Sidi Mohammed Ben Abdellah, Fez, Morocco (voucher specimen n° LERH-SC/15-03-21). Soxhlet extraction of 20 g of *Saussurea costus* (Falc) Lipsch was performed for 5 h in approximately 150 mL of solvents, resulting in 70% ethanol (organic) and distilled water (aqueous extract); the soxhlet extraction temperature regime heats the solvent to boiling temperature (>78–100 °C). The filtered extracts were then evaporated to dryness using a rotary evaporator under vacuum. These extracts were concentrated until dry and then the dry extracts were stored at a temperature of (2–6 °C). The extraction was carried out in triplicate.

### 2.2. Phytochemical Screening

Screening is a qualitative analysis technique that is based on staining and precipitation reactions that allows to determine beforehand the nature of the different constituents that are contained in the plants [24,25,26]. Screening was performed for the total extracts of *Saussurea costus* (Falc) Lipsch. The protocol that was followed for the screening of Saponosides:

Sterols and terpenes: 1 mL of acetic anhydride, 0.5 mL of chloroform, and 0.5 mL of concentrated sulfuric acid were added to 5 mL of each extract in a test tube without shaking, the appearance, in interphase, of a purple or violet ring, turning blue then green, indicates the presence of both sterols and terpenes [27].

Steroidal heterosides: we evaporated 10 mL of each extract in which was added 10 mL of anhydrous chloroform and then mixed with 5 mL of acetic anhydrous in which some sulfuric acid was added. The mixture was shaken and then left to rest. If the purplish coloration becomes green then that indicated the existence of steroidal heterosides [27].

Concerning the tannins, we put 1 mL of the extract in a test tube, to which a diluted solution of 1% FeCl_3_ was added. The development of a blackish blue or greenish coloration or the presence of a precipitate shows the presence of tannins [28].

For the screening of quinones, we put 1 mL of the extract in a test tube, to which a few drops of 1/10 NaOH were added. When the aqueous phase turns yellow, purple, or red testifies to the presence of quinones [28].

For Alkaloids, 2 mL of the extract was put into two test tubes (one mL each) to which 5 drops of Mayer’s reagent were added in the first tube and 5 drops of Wagner’s reagent in the second tube. The appearance of a precipitate indicates the presence of alkaloids [28].

A total of 5 mL of the extract was put in a test tube, to which was added 5 mL of hydrochloric alcohol (95% ethanol, HCl concentrated to equal parts in volumes), plus some magnesium chips to determine the existence or the absence of flavonoids. The appearance of a yellow-orange coloration indicates the presence of flavone [28].

### 2.3. In Vitro Study of Antilithiatic Activity

#### 2.3.1. Cystine Stone Dissolution Test

The purpose of this research is to see how *Saussurea costus* (Falc) Lipsch root extracts affect urinary cystine stone mass reduction over a six-week period while maintaining optimal physiological conditions (37 °C, 0.9 percent NaCl) [8].

The stones were obtained from the nephrology department of Hassan II University Hospital in Fez, Morocco. The Fourier transform infrared spectroscopy (FTIR) technique was used to determine the chemical composition of the stones [29]. We used Hannache’s approach to investigate the effect of aqueous and ethanolic extracts of this plant on cystine stones [8]. In 50 mL of physiological solution (NaCl 9 g/L), 0.5 % of plant extract was made; the 9 g/L NaCl solution was used as a negative control to examine changes in the stone mass and structure. As a positive control, potassium citrate was employed.

The stones were suspended in the extract at 37 °C in a permeable bag. For 6 weeks, each extract was subjected to continual magnetic stirring at 130 rpm. The pH of the solution was evaluated every 7 days with a pH meter for each experiment, and the mass loss of the kidney stones was determined by weighing the stone after drying in a 40 °C oven for 18 h. Scanning electron microscopy (SEM) was used to examine the surface of the stones before and after the experiment. Three times each experiment was carried out. The following formula was used to compute the percentage of mass loss % DR:

$$\mathrm{DR}\%=\frac{Wi\;-Wf}{Wi}$$

*W*i = the initial weight

*W*f = the final weight of calculi for each week.

##### The Sample Preparation (FTIR)

The analysis of the sample calculi was performed by dispersing 5% of the sample in 95% of KBr. The homogeneous powder resulting from this trituration was placed in a pellet mold with a diameter of 13 mm. The mold was put under pressure of 10 tons under a vacuum that was created using a pneumatic press This pressure was exerted during 2 to 3 min, then a translucent pellet of 1 mm thickness was obtained which was used for the analysis in the device, a Burker optic GMBH and CO.KG.Germany. The obtained pellet was carefully transferred into a pellet holder, which was then placed in the sample compartment in the path of the infrared radiation. The analysis was performed in a range of wavelengths between 4000 and 400 cm^−1^. We obtained the infrared spectrum which allows to eliminate the parasite absorbances (H_2_O and CO_2_) and to determine a spectrum with the characteristic absorption bands of the sample.

#### 2.3.2. Turbidity Test

The evaluation of the antilithiatic activity was studied by a turbidimetric method on calcium oxalate crystals. The inhibition of calcium oxalate crystallization was studied by measuring the optical density of solutions that were prepared by a mixture of dehydrated calcium chloride (solution A) (7 mmol/L) and sodium oxalate (solution B) (2 mmol/L), containing 200 mmol/L NaCl. In the absence and presence of plant extracts, in an ultraviolet-visible spectroscopy of type LABTRON LUS—Series Double Beam UV/Vis Spectrophotometer, United Kingdom, the optical density was determined at 620 nm [30].

1. Study in the absence of inhibitor

Crystallization started when 50 mL of a sodium oxalate solution was added to 50 mL of a calcium chloride solution. The mixture was incubated at 37 °C for 30 min. The optical density (OD) of the solution was read at 620 nm using an ultraviolet-visible spectrophotometer.

2. Study in the presence of inhibitor

Plant extracts were studied at different concentrations (0.5, 1.2 mg/mL) using sodium chloride. For each experiment, three replicates were performed. The percentage of inhibition I (%) that was produced by the plant extract was calculated by the following formula:

$$\%\mathrm{of}\;\mathrm{inhibition}=\frac{\mathrm{Control}\;\mathrm{absorbance}\;-\mathrm{Test}\;\mathrm{absorbance}}{\mathrm{Control}\;\mathrm{absorbance}}$$

Test absorbance = Absorbance in the presence of inhibitor (extract),

Control absorbance = Absorbance without inhibitor (negative control).

#### 2.3.3. In Vitro Crystallization Assay

The focus of this research was on microscopic observation. The effects of plant extracts on synthesized calcium oxalate crystals were investigated in the presence and absence of the extracts. We made a stock solution with 15 mL calcium chloride (8 mmol) and 15 mL sodium oxalate (1 mmol) containing 200 mmol NaCl in a volume of 30 mL, and added 5 mL sodium acetate (10 mmol) to get the pH to 5.7. Following crystal formation, 20 mL of *Saussurea costus* (Falc) Lipsch extract solutions (0.5 g, 1 g, and 2 g/L) were added to 20 mL of calcium oxalate solution. For 24 h, all the samples were agitated at 500 rpm at 37 °C. The morphology and crystal number of each sample were then determined using a light microscope (OLYMPUS U-SPT Japan) (400×) [1].

### 2.4. Antioxidant Activity

#### 2.4.1. DPPH Assay

In tubes, 750 microliters of a dilution series (2 mg/mL to 0.25 mg/mL) of each extract and 1.5 mL of the freshly prepared methanolic solution of DPPH (4 mg DPPH, dissolved in methanol) were introduced. The mixtures were vortexed and placed in the dark at room temperature for 30 min.

After incubation, the reading was performed by measuring the absorbance at a wavelength of λ = 517 nm. We also prepared the negative control which is composed of DPPH solution and methanol, the positive control is represented by ascorbic acid. The percentages of inhibition were calculated by the following relationship:

The antioxidant activity of our extracts was expressed as IC50, which is the concentration of the tested sample that is required to reduce 50% of the DPPH radical [27,31].

$$\%\;\mathrm{Of}\;\mathrm{antioxidant}\;\mathrm{activity}=\frac{\mathrm{AC}\;-\mathrm{AS}}{\mathrm{AC}}\times 100$$

AC: Absorbance of the negative control

AS: Absorbance of the sample

#### 2.4.2. Ferric Reducing Power Assay (FRAP)

A total of 1 mL of each extract was mixed with 2.5 mL of phosphate buffer (0.2 M, pH 6.6) and 2.5 mL of 1% (*w*/*v*) aqueous potassium ferricyanide (K_3_FeCN_6_) solution. The solutions were shaken immediately and mixed well, then they were incubated in a water bath at 50 °C for 20 min. Then, 2.5 mL of 10% (*w*/*v*) trichloroacetic acid was added to the reaction mixture. The whole solution was centrifuged at 3000 rpm for 10 min. At the end, 2.5 mL of the supernatant was mixed with 2.5 mL of distilled water and 0.5 mL of 0.1% (*w*/*v*) aqueous ferric chloride solution FeCl_3_ [31]. The results are expressed in milligrams of ascorbic acid equivalent per gram of extract. The absorbances were measured at a wavelength of 700 nm.

$$\mathrm{RP}=\frac{\mathrm{AC}\;-\mathrm{AS}}{\mathrm{AC}}\times 100$$

AC: Absorbance of the control

AS: Absorbance of the sample

### 2.5. Statistical Analyses

The data are presented as the mean values of three independent experiments (triplicate) and analyzed using a one-way (ANOVA). The values with (*p* < 0.05) were deemed significant. For statistical analysis, GraphPad Prism 7 and Microsoft Excel 2012 were used.

## 3. Results

### 3.1. Extraction Efficiencies

The solvents that were used revealed that the extraction yields varied from one extract to the next. The aqueous extraction of the plant yields more than the ethanolic extract. According to the results in Table 1, the aqueous plant extract has a higher yield (28.41%) than the ethanolic extract. The aqueous extract of the plant has a higher yield (28.41%) than the ethanolic extract. This is due to the fact that the aqueous extract is highly polar and is capable of extracting and dissolving polar molecules which increases the productivity of polar active metabolites in the extract. As demonstrated in this work, these polar active metabolites play a role in the dissolution of cystine stones.

### 3.2. Results of Phytochemical Screening

Preliminary analysis of the phytochemical composition of the roots of *Saussurea costus* (Falc) Lipsch revealed the presence of chemical classes, with the nature of the plant’s chemical compounds primarily consisting of tannins, quinones, sterols, and alkaloids (Table 2).

#### GC/MS profiling of *Saussurea costus* Aqueous and Ethanol Extracts

**Figure 1:**
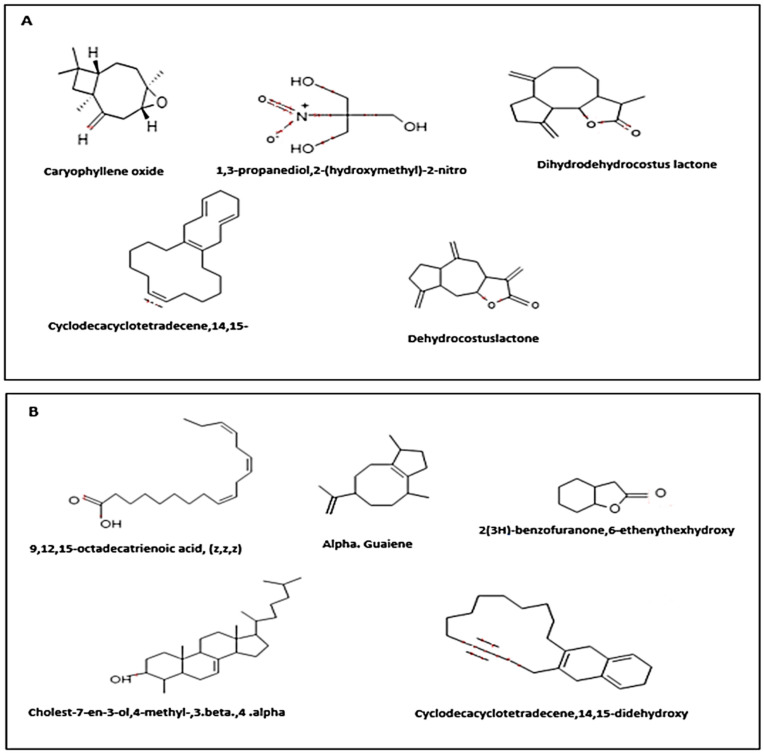
(**A**) Representative structures of the major metabolites that were identified in the ethanolic extract of the roots of *Saussurea costus*. (**B**) Representative structures of the main metabolites that were identified in the aqueous extract of the roots of *Saussurea costus*.

The roots of *Saussurea costus* (Falc) Lipsch are herbaceous plants belonging to the Asteraceae family and are widely used in traditional medicine for the treatment of various ailments. To expand our knowledge about this important medicinal plant, we used the GC/MS approach to determine the chemical constituents that are present in the aqueous and ethanolic extracts the plant of *Saussurea costus* (Falc) Lipsch. The results of GC/MS analysis of *Saussurea costus* (Falc) Lipsch that were obtained in the works [32,33,34,35,36] showed the main components with their retention times and relative percentage of the total peak area. They led to the discovery of 61 (Table 3) and 18 (Table 4) compounds in the ethanolic and aqueous extracts, respectively. Figure 1 represents the major compounds that were identified in the ethanolic and aqueous extracts.

### 3.3. In Vitro Study of Antilithiatic Activity

#### 3.3.1. Cystine Stone Dissolution Test

**Figure 2:**
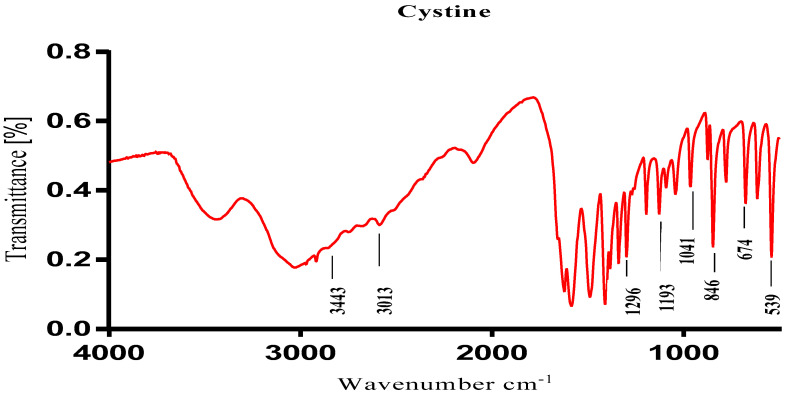
Spectrum (FT-IR) of a cystine stone.

The chemical composition of the kidney stones was identified using the Fourier transform infrared (FTIR) spectroscopy technique. The transmission spectrum is shown in (Figure 2). These results indicate that the type of stone is cystine which is characterized by the peaks 539.99 and 846.22 cm^−1^.

#### 3.3.2. Evolution of Mass Loss

**Figure 3:**
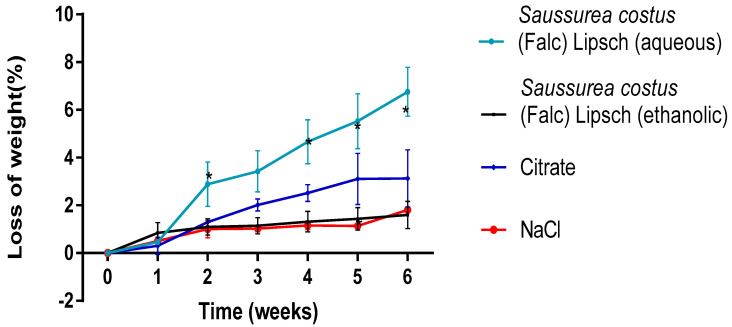
Evolution of the effect of extracts as a function of time on the loss of cystine stone mass (%) during 6 weeks. (Each value represents the average of three trials ± SD). * *p* value < 0.05.

Figure 3 depicts the evolution of cystine stone weight loss in the presence of aqueous and ethanolic extracts of *Saussurea costus* (Falc) Lipsch, positive control (Citrate) and negative control (NaCl). It can be seen that the percentage of stone dissolution increases with time between the first and last week. Indeed, a rate from (0.468 ± 0.100) to (6.756 ± 1.024) (*p* < 0.05) for the aqueous extract of *Saussurea costus* (Falc) Lipsch, from (0.306 ± 0.356) to (3.127 ± 1.204) (*p* < 0.05) for the ethanolic extract of *Saussurea costus* (Falc) Lipsch, from (0.306 ± 0.356) to (3.127 ± 1.204) (*p* < 0.05) for citrate, and from (0. 508 ± 0.136) to (1.809 ± 0.128) (*p* < 0.05) for NaCl.

#### 3.3.3. Evolution of pH

**Figure 4:**
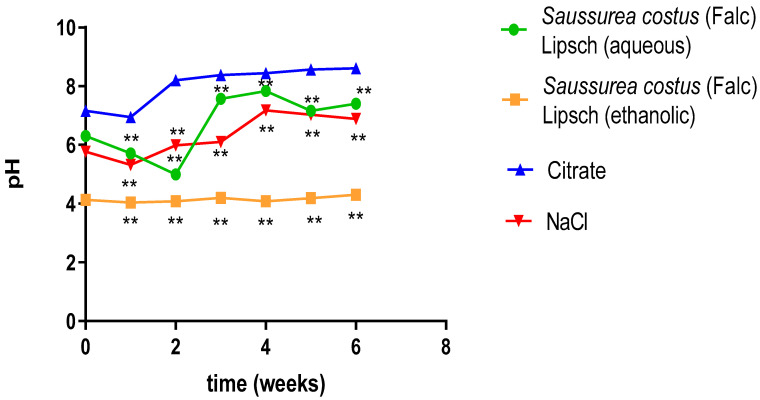
Evolution of the effect of extract type as a function of time on pH (each value represents the average of three trials ± SD). ** *p* value < 0.01.

Figure 4 depicts the evolution of the pH during the dissolution of cystine stones as a function of time in the presence of aqueous and ethanolic solutions of *Saussurea costus* (Falc) Lipsch. Citrate is a positive control and NaCl is a negative control. The outcome showed that the pH values is slightly acidic for the ethanolic extract of *Saussurea costus* (Falc) Lipsch with an increase from (5.71 ± 0.01) to (7.403 ± 0.005) (*p* < 0.05) and the pH values of aqueous extract of *Saussurea costus* (Falc) Lipsch are generally stable with a rate of (4.036 ± 0.0288) to (4.303 ± 0.066) (*p* < 0.05). We found from these results that dissolution is independent of pH of cystine calculi for ethanolic extract of *Saussurea costus* (Flac) Lipsch.

#### 3.3.4. Scanning Electron Microscopy

**Figure 5:**
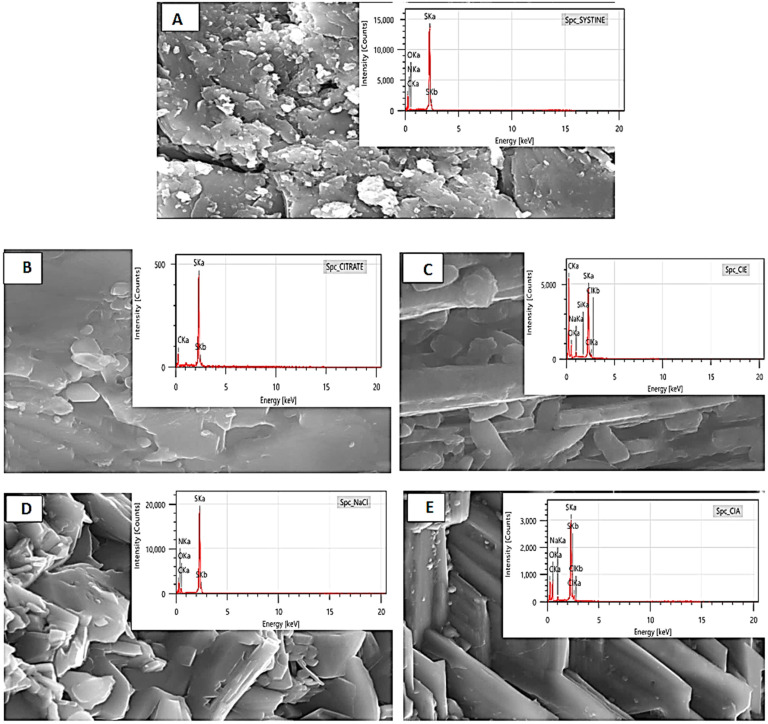
(**A**) The surface of the crystals visualized by SEM which is coupled to EDX before any treatment (**B**) The surface of the crystals visualized by SEM which is coupled to EDX after treatment by citrate (**C**) The surface of the crystals visualized by SEM which is coupled to EDX after treatment by the solution containing the ethanolic extract of *Saussurea costus* (Falc) Lipsch (**D**) The surface of the crystals visualized by SEM which is coupled to EDX after treatment by the aqueous solution of NaCl 9 gL^−1^ (**E**) The surface of the crystals visualized by SEM which is coupled to EDX after treatment by the solution containing the aqueous extract of *Saussurea costus* (Falc) Lipsch.

The results of analysis of cystine crystallites before and after treatment with ethanolic and aqueous extracts of *Saussurea costus* (Falc) Lipsch. Citrate is a positive control and NaCl is a negative control, respectively (Figure 5). The observed results showed an effect of aqueous and ethanolic extracts on the dissolution of cystine stones with the monitoring of changes in crystallite morphology to confirm the existence of interactions between the extracts of this plant and cystine stones.

### 3.4. Turbidity Test

#### 3.4.1. Crystal Synthesis

**Figure 6:**
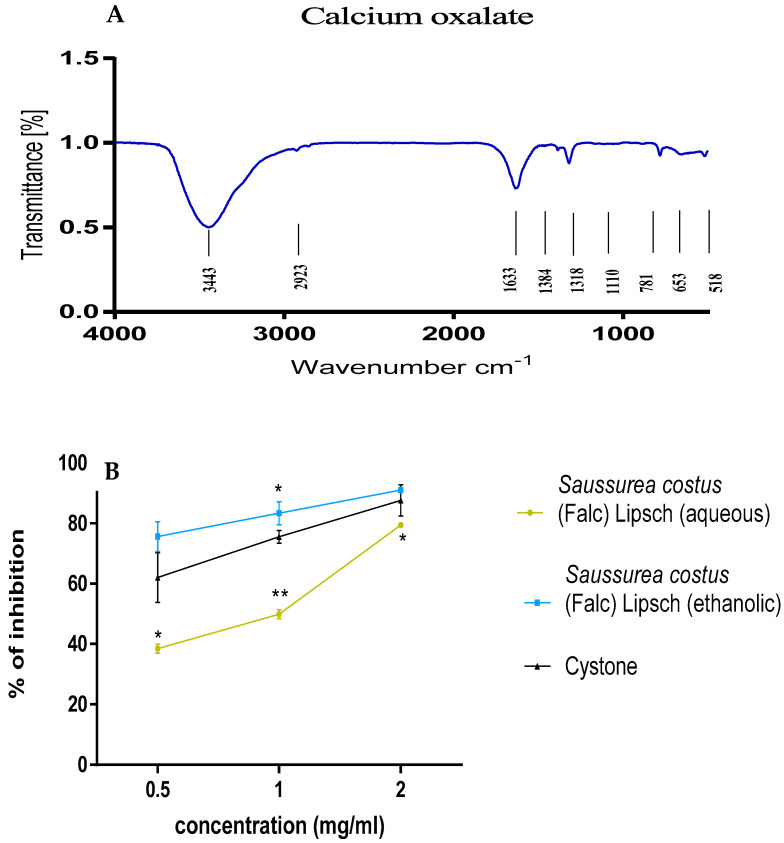
(**A**) Spectra of calcium oxalate, and (**B**) the effect of the extracts on the crystallization of calcium oxalate. * *p* value < 0.05, ** *p* value < 0.01.

The determination of the chemical constitution of the synthesized crystals were performed by Fourier transform infrared spectroscopy (FT−IR). The obtained spectrum is represented in Figure 6A. Figure 6B shows the evaluation of the percentage of inhibition of calcium oxalate by the turbidimetric method with different concentrations of extracts of roots of *Saussurea costus* (Falc) Lipsch, and the positive control of cystone. Analysis of the data in Figure 5B showed a high efficiency of the ethanolic extract compared to the aqueous extract in inhibiting calcium oxalate crystals. In addition, the percentage of inhibition of crystals is (91.017 ± 0.299) (*p* < 0.05) for the ethanolic extract of *Saussurea costus* (Falc) Lipsch of 2 mg/mL, followed by the aqueous extract of *Saussurea costus* (Falc) Lipsch with a percentage of (79.441 ± 0.45775) (*p* < 0.01).

#### 3.4.2. Crystallization Test by Microscopic Observation

We studied the evolution of crystal presence in the absence and presence of inhibitors using an optical microscope. The observation was made when materials were placed in a Malassez cell to calculate the number of crystals in mm^3^. The inhibitors of *Saussurea costus* (Falc) Lipsch extracts and the cystone solution as a positive control are shown in Table 5.

**Figure 7:**
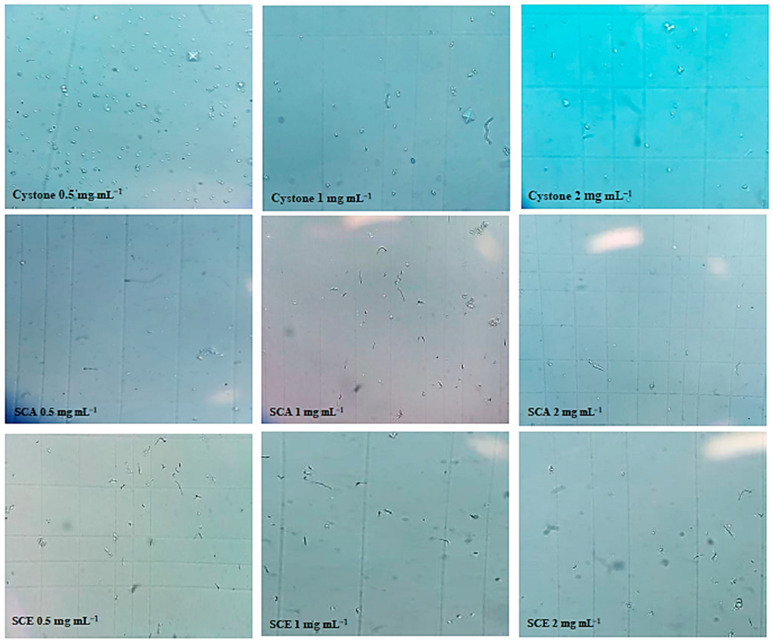
Microscopic observation of crystals at different inhibitor concentrations.

The number of crystals that were generated was dramatically reduced after adding ethanolic and aqueous extracts of *Saussurea costus* (Falc) Lipsch to the crystals for 24 h (Figure 7). The number of crystals between concentrations of 0.5 and 2 mg/mL is found to decrease from 300 to 250 for the ethanolic extract and from 500 to 200 for the aqueous extract using an optical microscope (400×).

### 3.5. Antioxidant Activity

#### 3.5.1. DPPH

**Figure 8:**
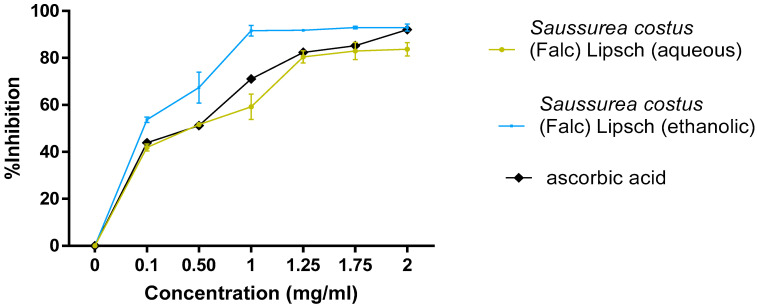
Antiradical activity (DPPH) of extracts (each value represents the average of three trials ± SD).

The results of the anti-free radical activity that was tested by DPPH are represented by the percentages of inhibition for each concentration as well as the values of the concentration IC_50_ (Figure 8). The ethanolic extract of *Saussurea costus* (Falc) Lipsch presents a better antiradical activity of IC_50_ = 0.12325 mg mL^−1^ (*p* < 0.05) in comparison with ascorbic acid (IC_50_ = 0.2142795 mg mL^−1^) (*p* < 0.05).

#### 3.5.2. FRAP

**Figure 9:**
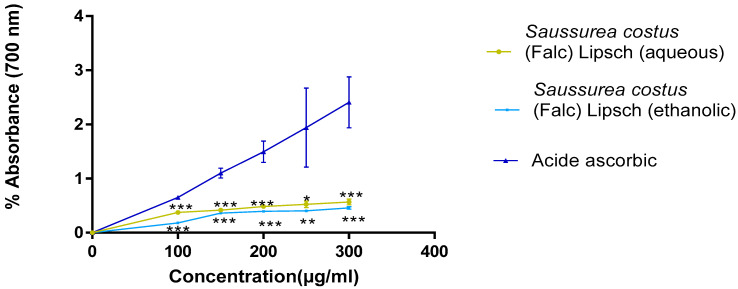
Evaluation of antioxidant activity of extracts by FRAP method (each value represents the average of three trials ± SD). * *p* value <0.05, ** *p* value <0.01, *** *p* value <0.005.

From the results that were obtained in Figure 9, it can be seen that the reducing power of the extracts of *Saussurea costus* (Falc) Lipsch is that the reducing power of iron is proportional to the increase in the concentration of the extracts. The aqueous and ethanolic extract of *Saussurea costus* (Falc) Lipsch presents a better reducing power of OD = 0.56 ± 0.05 and of OD = 0.39 ± 0.01, respectively.

## 4. Discussion

The results that are presented in the Table 1 show that the aqueous plant extract has a higher yield (28.41% ± 0.01) compared to the ethanolic extracts. Phytochemical screening (Table 2) revealed the presence of several phytochemical classes (sterols and terpenes, steroidal heterosides, flavonoids, tannins, quinones, alkaloids) in the aqueous and ethanolic extracts of *Saussurea costus* (Falc) Lipsch, except that steroidal heterosides are absent in the extracts. Other studies reported that the flavonoids are present in the ethanolic extract and absent in the aqueous extract [37,38].

Regarding the test results of cystine stone dissolution, the chemical composition of kidney stones was identified using the Fourier transform infrared spectroscopy (FTIR) technique. Analysis of the FT-IR spectra showed the presence of band at 3013.49 cm^−1^ of acid OH. A band of primary amine N-H was observed at 3443.76 cm^−1^. Absorption bands of acid C-O observed at 1193.48 cm^−1^ and at 1296.52 cm^−1^. An absorption band was observed at 1041.16 cm^−1^ of C-N. Absorption bands of C-H of alkanes were observed at 1485.88 cm^−1^ and at 1413.13 cm^−1^. An absorption band was observed at 1041.16 cm^−1^ of C-N corresponding to the primary amine. A band of C-S of sulfide was observed at 674.85 cm^−1^ [39]. FT-IR spectrum analysis showed and confirmed that the type of compound is cysteine [40].

The mass loss of cystine stones is significantly high for the aqueous extract of *Saussurea costus* (Falc) Lipsch roots (*p* < 0.05). On the other hand, the ethanolic extract of both plants are not significant (*p* < 0.05). The results we obtained are less important compared to those that were found by Hannache [8]. The plants *Arenaria ammophila* L and *Parietaria officinalis* L that were studied in this work showed that the percentage of dissolution of cystine is 64% which is significant compared to the plant that we examined which represents a percentage of dissolution of 6%. The lack of efficiency of our plant may be due to the method of extraction and, therefore, to the compounds that are present in the extract. pH monitoring showed a significant variation (*p* < 0.01) for all the extracts.

The results in Figure 5 showed a significant effect of aqueous and ethanolic extract on the dissolution of cystine kidney stones with the monitoring of the changes in the morphology of crystallites to confirm the existence of interactions between the extracts of this plant and cystine stones. These results show that before the treatment, the cystine stones constituted of C, N, O, and S which confirms the results that were obtained in the infrared spectroscopy (Figure 2). On the other hand, after the treatment with these aqueous and ethanolic extracts, we found that cystine constituted of O, N, S, and Cl for the aqueous extract of *Saussurea costus* (Falc) Lipsch; by C, O, Na, S, and Cl for the ethanolic extract of *Saussurea costus* (Falc) Lipsch; and by C and S for citrate. Changes in the ultrastructural characteristics and constituent atoms of cystine stones after treatment were confirmed by SEM-EDX technique. The results suggest that this plant exerts a curative effect which may be due to the presence several chemical compounds in these extracts.

The analysis of the FT-IR spectra (Figure 6A) confirmed the presence of band at 3443.87 cm^−1^ the OH stretching of water. An out-of-plane C-O band and another O-C-O in-plane bending band was observed at 781.03 and 518.06 cm^−1^, respectively, indicating the presence of calcium oxalate monohydrate crystals [29]. The absorption bands that were observed at 1633 cm^−1^ and 1318 cm^−1^ were attributed to the antisymmetric carboxyl stretching band (vas (COO-)) and symmetric stretching band (vs (COO-)); these bands correspond to dehydrated calcium oxalate crystals. An absorption band that was observed at 1384 cm^−1^ (COO-) corresponds to calcium oxalate trihydrate crystals [39]. The FT-IR spectrum analysis showed that the crystals that we synthesized are calcium oxalate dihydrate crystals with the presence of traces of calcium oxalate monohydrate and trihydrate crystals. The results that were obtained in (Figure 6B) indicated that the ethanolic extract of *Saussurea costus* (Falc) Lipsch has a significant effect (*p* < 0.05). On the other hand, the aqueous extract of *Saussurea costus* (Falc) Lipsch is not significant (*p* < 0.01).

When it comes to microscopic crystallization in Figure 7, it is observed that as the concentration of the plants increases, the number of crystals decreases. This confirms the results that were obtained in the turbidity test which shows that the ethanolic extracts of *Saussurea costus* (Falc) Lipsch are effective against calcium oxalate and are also able to prevent the formation of CaOx crystals; these results are, therefore, similar to those that were found in the study that was conducted by Li et al. [1].

Regarding the antioxidant activity, Figure 8 proves that the ethanolic extract of *Saussurea costus* (Falc) Lipsch presents a better antiradical activity of (IC_50_ = 0.12 mg mL^−1^) in comparison with ascorbic acid (IC_50_ = 0.21 mg mL^−1^). On the other hand, the aqueous extract showed a weak antiradical activity compared to the values of ascorbic acid. The results that we obtained are consistent with those that were obtained in the work done by Pandey et al. [41]. This good antioxidant activity of the ethanolic extract can be explained by the nature of the flavonoid compounds that are present in these extracts.

The results that were obtained (Figure 9) showed that the capacity of *Saussurea costus* (Falc) Lipsch extracts to reduce iron is much lower than that of ascorbic acid. At the concentration of 300 μg/mL, the reducing power is much higher in the aqueous extract of *Saussurea costus* (Falc) Lipsch with (OD = 0.56 ± 0.05) compared to the ethanolic extract of this plant with (OD = 0.39 ± 0.01). The results that we obtained are consistent with those that were obtained in the work done by Singh et al. [42]. The reducing power of the plant extracts is probably due to the presence of hydroxyl group in the tannin compounds which can serve as donor electron.

## 5. Conclusions

According to phytochemical screening results, *Saussurea costus* (Falc) Lipsch contains a lot of flavonoids, tannins, quinones, alkaloids, sterols, and terpenes. These extracts of *Saussurea costus* (Falc) Lipsch in the present work have shown an effect on cystine stone dissolution; the effect of this plant was tested using scanning electron microscopy. The most effective extract was an aqueous extract of *Saussurea costus* (Falc) Lipsch. The ethanolic extract of this plant has a significant dose-dependent inhibitory activity on oxalocalcic crystallization, according to turbidimetric. This antilithiatic effect could be attributed to the high concentration of biologically active substances in this plant. The free radical scavenging test results revealed that the ethanolic extract of *Saussurea costus* (Falc) Lipsch has higher DPPH method free radical scavenging activity. Additionally, the aqueous extract of *Saussurea costus* (Falc) Lipsch has higher FRAP method reducing power. It would be critical to put these extracts to the test in vivo. A study of this plant’s toxicity is also recommended.

## Figures and Tables

**Table 1:** The results of the extraction yields of the plants.

The Plants	The Extracts	The Yields%
*Saussurea costus* (Falc) Lipsch	Ethanolic extract	18.79 ± 0.01
*Saussurea costus* (Falc) Lipsch	Aqueous extract	28.41 ± 0.01

**Table 2:** Results of the phytochemical screening.

*Saussurea costus* (Falc) Lipsch	Sterols and Terpenes	Steroidal Heterosides	Flavonoids	Tannins	Quinones	Alkaloids
Aqueous extract	+	-	-	+	+	+
Ethanolic extract	+	-	+	+	+	+

+: a substance found in the extract. -: a substance not found in the extract.

**Table 3:** The retention time (RT) and peak area (percent) of the different compounds that were found in an ethanolic extract of *Saussurea costus* that were analyzed by GC-MS.

Chemical Constituents	Molecular Formula	RT (min)	Peak Area %	Molecular Weight
Dehydrocostuslactone	C_15_H_18_O_2_	30.41	49.68	230
Saussurea lactone	C_15_H_22_O_2_	23.26	4.16	234
Costunolide	C_15_H_20_O_2_	26.31	3.59	232
Dihydrodehydrocostus lactone	C_15_H_18_O_2_	29.21	16.34	230
Beta-costol	C_15_H_24_O	25.55	2.74	220
(+)-Isovalencenol	C_15_H_24_O	31.71	2.52	220
Caryophyllene oxide	C_15_H_24_O	27.02	11.03	220
(+)-Isovalencen	C_15_H_24_O	31.71	2.52	220
Linoleic acid, methyl ester	C_19_H_34_O_2_	32.1	2.15	294
9-Octadecenoic acid, methyl ester	C_19_H_36_O_2_	32.22	1.54	296
Aristol-1(10)-en-9-ol	C_15_H_24_O	25.67	1.41	220
Hexadecanoic acid,methyl ester	C_17_H_34_O_2_	28.89	0.85	270
Valerenol	C_15_H_24_O	30.64	0.46	220
Reynosin	C_15_H_20_O_3_	31.05	0.39	248
Beta-caryophyllene oxide	C_15_H_24_O	21.4	0.38	220
Linolein, 2-mono-	C_21_H_38_O_4_	19.07	0.38	354
10-Heptadecen-8-ynoic acid, methyl ester, (E)-	C_18_H_30_O_2_	32.44	0.25	278
Octadecanoic acid, methyl ester	C_19_H_38_O_2_	32.71	0.24	298
16-Methyloxacyclohexadeca-3,5-dien-2-one	C_16_H_26_O_2_	32.81	0.24	250
Santamarine	C_15_H_20_O_3_	28.43	0.18	248
Glycidyl oleate	C_21_H_38_O_3_	33.39	0.22	338
Farnesene epoxide, E-	C_15_H_24_O	33.49	0.17	220
Eudesm-4(14)-en-11-ol	C_15_H_26_O	20.62	0.16	222
Trans-á-Ionone	C_15_H_26_O	38.52	0.15	222
Glycerin	C_3_H_8_O_3_	3.269	0.87	92
Ethoxyacetaldehyde diethylacetal	C_8_H_18_O_3_	3.346	1.28	162
2-Furanmethanol	C_5_H_6_O_2_	3.631	0.28	98
Methanamine, N-hydroxy-N-methyl-	C_2_H_7_NO	4.234	2.31	61
2-hydroxy-2-Cyclopenten-1-one	C_5_H_6_O_2_	4.754	0.31	98
2,4-Dihydroxy-2,5-dimethyl-3(2H)-furan-3-one	C_6_H_8_O_4_	5.715	0.29	144
2,5-Hexanedione	C_6_H_10_O_2_	7.066	0.21	130
Pentanoic acid,4-oxo- (Levulinic acid)	C_5_H_8_O_3_	7.154	0.11	116
2,5-Dimethyl-4-hydroxy-3(2H)-furanone (Furaneol)	C_6_H_8_O_3_	7.397	0.09	128
Maltol	C_6_H_6_O_3_	7.845	0.73	126
4H-Pyran-4-one,2,3-dihydro-3,5-dihydroxy-6-methyl-	C_6_H_8_O_4_	9.209	1.14	144
5-Hydroxymethylfurfural	C_6_H_6_O_3_	11.033	2.15	126
1,2,3-propanetriol,1-acetate	C_5_H_10_O_4_	11.343	1.53	134
Propanoic acid,3-(acetyloxy)-2-(hydroxymethyl)-,ethyl ester	C_8_H_14_O_5_	12.297	1.04	190
1,3-propanediol,2-(hydroxymethyl)-2-nitro-	C_4_H_9_NO_5_	15.686	11.05	151
11,11-Dimmethyl-spiro(2,9)dodeca-3,7-dien	C_14_H_22_	17.997	0.19	190
1,2,3,5-Cyclohexanetetrol, (1.alpha, 2.beta.3.alpha, 5.beta.)-	C_6_H_12_O_4_	18.585	0.76	148
9,12,15-octadecatrienoic acid,(z,z,z)	C_18_H_30_O_2_	19.174	2.49	278
Androstan-17-one,3-ethyl-3-hydroxy-,(5.alpha)-	C_12_H_34_O_2_	19.255	3.01	210
2-(4a,8-Dimethyl-1,2,3,4,4a,5,6,7-octahydro-naphthalen-2-	C_15_H_24_	20.604	0.31	204
Bicyclo(5.2.0)nonane,4-methylene-2,8,8-trimethyl-2-vinyl-	C_15_H_24_	20.677	0.32	204
Andrographolide	C_20_H_30_O_5_	20.777	0.17	350
Bicyclo(5.3.0)decane,2-methylene-5-(1-methylvinyl)-8-	C_15_H_24_	20.927	4.36	204
Gamma.-guarjumenepoxide-(2)	C_15_H_24_O	20.993	1.77	216
2(3H)-benzofuranone,6-ethenylhexahydro-6-methylene-7-	C_15_H_20_O_2_	21.855	3.88	232
Alloaromadendrene	C_15_H_24_	22.237	1.36	204
Pentadecanoic acid	C_15_H_30_O_2_	23.156	1.79	242
4,7,10,13,16,19-Docosahexaenoic acid, methyl ester	C_23_H_34_O2	23.227	3.18	342
Cyclodecacyclotetradecene,14,15-didehydro-	C_22_H_32_	23.933	39.59	296
Beta.-Guaiene	C_15_H_24_	25.734	1.21	204
Isosteviol methyl ester	C_21_H_32_O_3_	25.863	3.97	332
Bufa-20,22-dienolide,14,15-epoxy-	C_24_H_34_O_2_	26.180	5.91	354
-Norlupan-28-oic acid,3-hyroxy-21-	C_29_H_46_O_4_	26.997	1.05	458
Octadecanoic acid,2,3-dihydroxypropyl-	C_21_H_42_O_4_	28.511	0.41	358
9,12-Octadecadienoic acid (z,z)-	C_18_H_32_O_2_	29.991	0.34	280
Pregnennolone	C_21_H_32_O_2_	30.631	0.21	316
Spiro(5.5)undeca-1,8-diene,1,5,5,-	C_15_H_24_	31.009	0.33	204

**Table 4:** The retention time (RT) and peak area (percent) of the different compounds that were found in an aqueous extract of *Saussurea costus* that were analyzed by GC-MS.

Chemical Constituents	Molecular Formula	RT (min)	Peak Area %	Molecular Weight
2-Cyclopenten-1-one,2-hydroxy-	C_5_H_6_O_2_	4.772	0.61	90
1-Dodecanol	C_12_H_26_O	10.071	0.36	186
1,2,3-propanetriol,1-acetate	C_5_H_10_O_4_	11.401	1.16	134
Cyclohexane,1-ethenyl-1-methyl-2,4-bis-	C_15_H_24_	14.314	0.17	204
Caryophyllen	C_15_H_24_	14.918	0.15	204
3-Buten-2-one,4-(2,6,6-trimethyl-2-cyclo-	C_13_H_20_O	15.000	0.18	192
1,3-propanediol,2-(hydroxymethyl)-2-	C_4_H_9_NO_5_	15.220	2.88	151
9,12,15-octadecatrienoic acid,(z,z,z)-	C_18_H_30_O_2_	19.178	3.47	270
Cyclohexane,1,2-diethenyl-4-(1-methyle-	C_13_H_20_	19.256	3.12	176
Alpha.-Guaiene	C_15_H_24_	20.928	4.17	204
Delta.4-androstene-3.beta,17.beta.-diol	C_19_H_30_O_2_	20.993	1.88	290
2(3H)-benzofuranone,6-ethenythexhydroxy-	C_15_H_20_O_2_	21.842	4.42	232
4,7,10,13,16,19-Docosahexaenoic acid, methyl ester	C_22_H_34_O_2_	23.227	2.96	296
Cyclodecacyclotetradecene,14,15-didehydroxy	C_22_H_32_	23.889	61.69	296
1,4-Methanocycloocta(d)pyridazine,1,4,4a-	C_12_H_20_N_2_	25.863	2.73	192
3-oxatricyclo(20.8.0.0(7,16))triaconta-1-	C_29_H_42_O	26.178	3.49	406
Octadecanoic acid,2,3-dihydroxypropyl-	C_21_H_42_O_4_	28.520	1.44	358
Cholest-7-en-3-ol,4-methyl-,(3.beta, 4.alpha-	C_28_H_48_O	31.285	5.10	400

**Table 5:** The number of crystals in the presence of extracts of *Saussurea costus* (Falc) Lipsch and cystone solution.

Concentration	Cystone Solution	*Saussurea costus* (Falc) Lipsch (Aqueous)	*Saussurea costus* (Falc) Lipsch (Ethanolic)
0.5 mg/mL	1000 mm^3^	900 mm^3^	600 mm^3^
1 mg/mL	700 mm^3^	400 mm^3^	300 mm^3^
2 mg/mL	500 mm^3^	300 mm^3^	250 mm^3^

## Data Availability

Not applicable.
